# Integrated Analyses Reveal Overexpressed Notch1 Promoting Porcine Satellite Cells’ Proliferation through Regulating the Cell Cycle

**DOI:** 10.3390/ijms19010271

**Published:** 2018-01-16

**Authors:** Yiren Jiao, Bo Huang, Yu Chen, Guangliang Hong, Jian Xu, Chingyuan Hu, Chong Wang

**Affiliations:** 1 National Engineering Research Center for Breeding Swine Industry, Guangdong Provincial Key Lab of Agro-Animal Genomics and Molecular Breeding, College of Animal Science, South China Agricultural University, Guangzhou 510642, China; yangzhuxiaozi005@163.com (Y.J.); huangbo@stu.scau.edu.cn (B.H.); chenyudk320@163.com (Y.C.); hgl2014@stu.scau.edu.cn (G.H.); xj30380226@163.com (J.X.); 2 Department of Human Nutrition, Food and Animal Sciences, College of Tropical Agriculture and Human Resources, University of Hawaii at Manoa, 1955 East-West Road, AgSci. 415J, Honolulu, HI 96822, USA; chinghu@hawaii.edu

**Keywords:** N1ICD, muscle satellite cells, proliferation, mRNA-seq, miRNA-seq, pig

## Abstract

Notch signaling as a conserved cell fate regulator is involved in the regulation of cell quiescence, proliferation, differentiation and postnatal tissue regeneration. However, how Notch signaling regulates porcine satellite cells (PSCs) has not been elucidated. We stably transfected Notch1 intracellular domain (N1ICD) into PSCs to analyze the gene expression profile and miRNA-seq. The analysis of the gene expression profile identified 295 differentially-expressed genes (DEGs) in proliferating-N1ICD PSCs (P-N1ICD) and nine DEGs on differentiating-N1ICD PSCs (D-N1ICD), compared with that in control groups (P-Control and D-Control, respectively). Analyzing the underlying function of DEGs showed that most of the upregulated DEGs enriched in P-N1ICD PSCs are related to the cell cycle. Forty-four and 12 known differentially-expressed miRNAs (DEMs) were identified in the P-N1ICD PSCs and D-N1ICD PSCs group, respectively. Furthermore, we constructed the gene-miRNA network of the DEGs and DEMs. In P-N1ICD PSCs, miR-125a, miR-125b, miR-10a-5p, ssc-miR-214, miR-423 and miR-149 are downregulated hub miRNAs, whose corresponding hub genes are marker of proliferation Ki-67 (MKI67) and nuclear receptor binding SET domain protein 2 (WHSC1). By contrast, miR-27a, miR-146a-5p and miR-221-3p are upregulated hub miRNAs, whose hub genes are RUNX1 translocation partner 1 (RUNX1T1) and fibroblast growth factor 2 (FGF2). All the hub miRNAs and genes are associated with cell proliferation. Quantitative RT-PCR results are consistent with the gene expression profile and miRNA-seq results. The results of our study provide valuable information for understanding the molecular mechanisms underlying Notch signaling in PSCs and skeletal muscle development.

## 1. Introduction

Satellite cells as a primary source of muscle progenitors were initially identified in the frog skeletal muscle by Mauro in 1961 [1]. Satellite cells locating a niche between the basal lamina and plasma membrane of mature myofibers play an indispensable role in supporting muscle growth, maintenance and regeneration of postnatal skeletal muscle [2,3,4,5]. Numerous studies have shown that satellite cells are usually quiescent in adult muscles [6,7,8]. In response to muscle injury, satellite cells become activated and re-enter the cell cycle to proliferate and differentiate. Notch signaling played an important role in the regeneration of injured muscle [9].

The Notch signaling pathway is one of the major regulatory pathways in myogenesis [9,10,11,12]. There are four Notch receptors (Notch1–Notch4), which are activated after binding to its ligands, such as Jagged 1, Jagged 2, Delta-like 1, Delta-like 3 and Delta-like 4 in mammals [13]. Once bound, Notch undergoes protease cleavage to free Notch intracellular domain (NICD) and translocates into nucleus, where it interacts directly with the transcription factor RBPJ (also known as CBF1, recombination signal binding protein for immunoglobulin kappa J region) to activate its target genes, such as hes related family bHLH transcription factor with YRPW motif (HEY) and HES family [14]. Notch1–3, HES1, HES2, HES5, HEY1 and hes related family bHLH transcription factor with YRPW motif-like (HEYL) were highly expressed in the quiescent satellite cells, which implies that Notch signaling is essential for maintenance of satellite cell quiescence. Notch is downregulated when satellite cells are activated [15,16,17]. Furthermore, the expression of Notch signaling is decreased in the quiescent mice satellite cells with RBPJ defect, which facilitated the spontaneous activation of satellite cells and allowed them to undergo terminal differentiation, thereby resulting in a severe depletion of satellite cells’ pool [15,16]. Knocking down Notch1 reduces PSCs (porcine satellite cells) proliferation, and overexpressing Notch1 with recombinant human NF-κB (nuclear factor kappa B) protein promotes PSCs proliferation though influencing cell cycle progression [18]. Furthermore, activation of Notch signaling promotes proliferation in mice satellite cells [10,19,20]. There exist two waves of Notch signaling during the activation of satellite cells. The first wave of Notch signaling maintains the quiescent state of the satellite cells. The second wave of Notch signaling rises after satellite cells are activated [4]. This explanation is supported by Notch expression being decreased at first and then increasing during muscle injury [9,16]. Furthermore, Notch signaling is also related to satellite cell differentiation. Due to the crosstalk between Notch and Wnt cascade via GSK3β, the inhibition of Notch signaling would be suitable for a rapid temporal switch to suppress Notch signaling and activate Wnt signaling, which facilitated satellite cell differentiation and fusion of myoblasts to myotubes [20]. Still, how the Notch signaling pathway regulates skeletal muscle myogenesis is not clear.

Small noncoding RNAs about 22 nucleotides long in the animal are classified as microRNAs (miRNAs), which regulate the expression of posttranscriptional protein-coding genes through interacting with the 3′-untranslated region (3′-UTR) of their target mRNAs [21]. Currently, 382 hairpin precursor miRNAs and 411 mature miRNAs have been identified in the miRBase database (Version 21), which is still far less than by comparing with human 2588 miRNA (http://mirbase.org/index.shtml). Numerous studies demonstrated that miRNA play an indispensable role in regulating muscle development [22]. In skeletal muscle, highly expressed miRNAs are called myomiRs, which include miR-1, miR-133a, miR-133b, miR-206, miR-208a, miR-208b, miR-486 and miR-499 [23]. Besides the myomiRs, there are also many miRNAs regulating satellite cell myogenesis, for example miR-31 [24]; miR-27a [25,26], miR128 [27], miR-221/miR-222 [28], miR-682 [29] and miR-181 [30]; miR-186 [31], miR-24 [32], miR-214 [33,34], miR-26a [35], miR-29b/c [36], miR-322/424 [37] and miR-503 [37] have been identified to regulate the quiescence, proliferation and differentiation stage of satellite cells respectively. Both Notch signaling and miRNA have been identified to play critical roles in regulating myogenesis, and four miRNAs, miR-1, miR-206, miR-146a and 146b, have been reported to regulate skeletal muscle myogenesis via Notch signaling [38,39,40,41]. However, none of these four miRNAs were studied in porcine skeletal muscle.

In this study, we hypothesized that the Notch signaling pathway might contribute to PSCs’ proliferation and differentiation by regulating specific genes and miRNAs. We used next generation sequencing technology (NGST) to characterize mRNA-seq and miRNA-seq of control and stably transfected with N1ICD in PSCs. We have identified many differentially-expressed genes (DEGs) and differentially-expressed microRNAs (DEMs) by using bioinformatics analysis. We have also established relations between these DEGs and DEMS to identify the miRNA-mRNA pairs that may be connected to the Notch signaling in PSCs’ development. These findings could provide a reference for further research of molecular mechanisms underlying Notch signaling in PSCs.

## 2. Results

### 2.1. Construction and Effect of a N1ICD Overexpressing Cell Line

**Figure 1:**
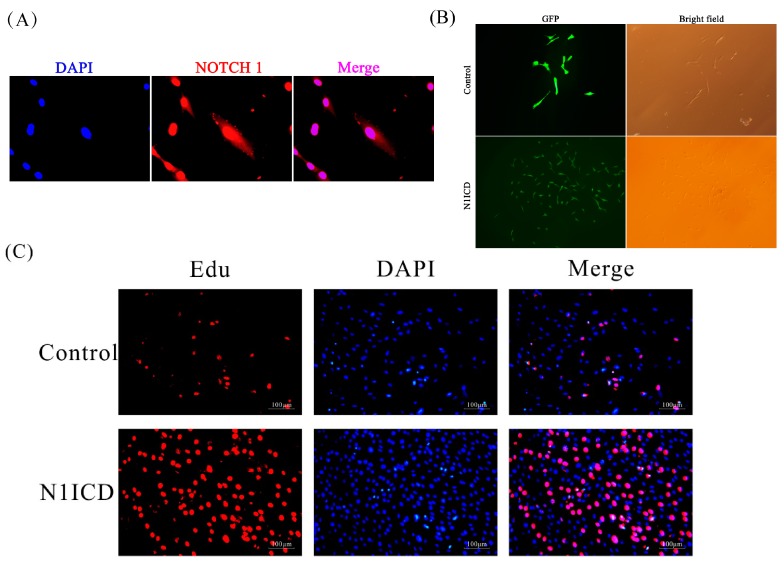

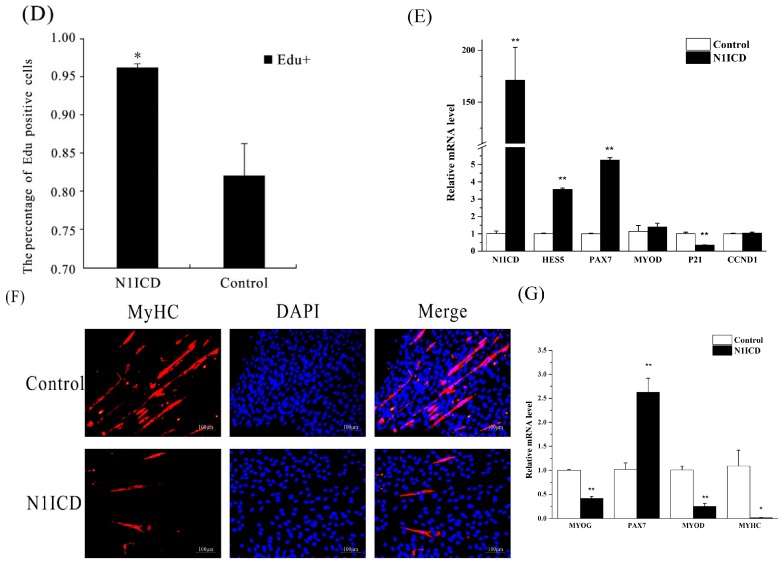
The model of N1ICD overexpressed PSCs. (**A**) The expression of N1ICD was tested by immunocytochemistry. DAPI, blue, represents nuclei; NOTCH1, red; Merge, pink, represents N1ICD expressed in nuclei of PSCs. (**B**) One week later, the degree of green fluorescence protein (GFP) in the control group is greener than the N1ICD-overexpressed group. However, the level of N1ICD expression in the N1ICD-overexpressed group is more than the control group. (**C**,**D**) Representative images of the immunofluorescent staining for proliferating PSCs are shown. Proliferating PSCs were labeled with Edu fluorescent dye (red). (**E**) Q-RT-PCR showed the changes of N1ICD, hes family bHLH transcription factor 5 (HES5), paired box 7 (PAX7), myogenic differentiation 1 (MYOD), cyclin dependent kinase inhibitor 1A (P21) and cyclin D1 (CCND1) in proliferating PSCs. Overexpressed N1ICD in PSCs did not show any changes in mRNA level of MYOD and CCND1. * *p* < 0.05; ** *p* < 0.01. Data are the mean ± S.E.M, *n* = 3 for each treatment. (**F**) Representative images of the immunofluorescent staining for differentiating PSCs are shown. Myosin heavy chain (MYHC) was labeled by fluorescent dye (red). Scale bar = 20 μm (×200 magnification). (**G**) Q-RT-PCR showed the changes of myogenin (MYOG), PAX7, MYOD and MYHC in overexpressed N1ICD differentiating PSCs. MYOG and MYOD significantly decreased while PAX7 significantly increased in overexpressed N1ICD differentiating PSCs. * *p* < 0.05; ** *p* < 0.01. Data are the mean ± S.E.M, *n* = 3 for each treatment.

Immunocytochemistry was performed to verify the distribution of Notch1 in PSCs. The result showed that N1ICD was highly expressed in the nucleus of PSCs (Figure 1A). We used green fluorescence to label the N1ICD vector to verify the model of stable overexpression of N1ICD in PSCs. After stably transfected to PSCs, the green fluorescence in the pEGFP-N1ICD group was higher than the control group, but the degree of green fluorescence was lower (Figure 1B). The Edu staining method was used to examine the effect of overexpression of N1ICD on PSCs proliferation. Twenty-four hours later, overexpressed N1ICD increased the percentage of Edu positive cells (*p* < 0.05, Figure 1C,D). Q-RT-PCR results showed that we have successfully overexpressed N1ICD in PSCs (*p* < 0.01, Figure 1E). Meanwhile, overexpressed N1ICD increased HES5, which is a downstream gene of Notch1 (*p* < 0.01, Figure 1E). The mRNA expression of paired box 7 (PAX7) was also increased, but the relative expression of cyclin dependent kinase inhibitor 1A (P21) was decreased (*p* < 0.01, Figure 1E).

Two groups of cells in six-well cell culture plates were induced to differentiation to examine the effect of overexpressed N1ICD in PSCs. The result showed that overexpressed N1ICD reduced the expression of MYHC compared with the control, and the number of myotubes was also significantly decreased (Figure 1F). Besides, the relative expression of myogenic differentiation 1 (MYOD) and myogenin (MYOG) was significantly decreased while Pax7 was increased (*p* < 0.01, Figure 1G). Furthermore, the relative expression of myosin heavy chain (MYHC) was decreased in the N1ICD overexpressed group (*p* < 0.05, Figure 1G).

All these results indicate that N1ICD was overexpressed successfully in the PSCs, and the elevated N1ICD promoted PSCs proliferation, but inhibited PSCs differentiation.

### 2.2. Characterization of mRNA and miRNA Transcriptome Sequencing Data

**Figure 2:**
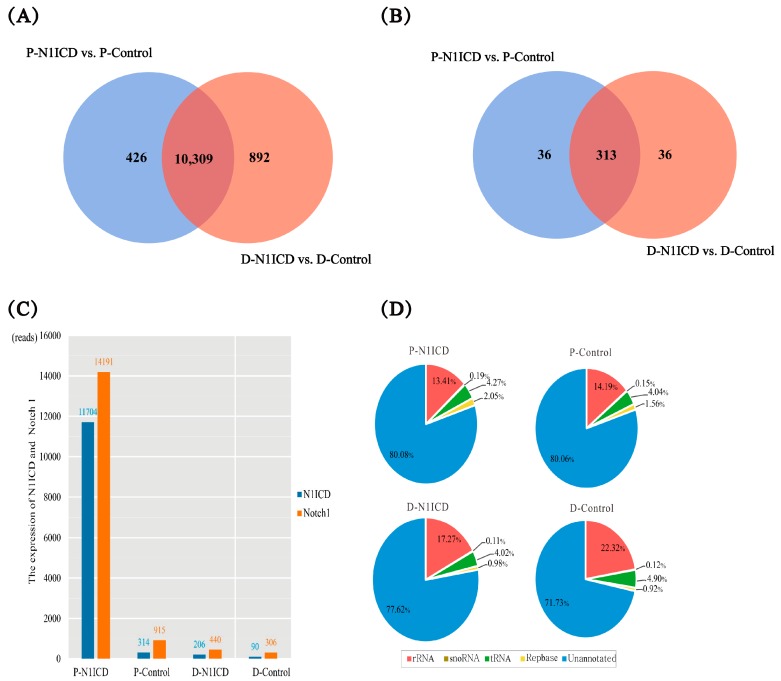
Gene and miRNA screening. (**A**) Number of genes expression; (**B**) Number of miRNAs expression; (**C**) Expression of N1ICD and Notch1 estimated by BLAST (Basic Local Alignment Search Tool); (**D**) Classification of the annotation of small RNA.

By using high-throughput mRNA sequencing, we have obtained about 55 million clean reads (50 base single-end reads) from four mRNA samples, an average of 13.7 million per each, and Q30 quality scores of all sample reads were greater than 85.81% by using fastQC (Table 1). Approximately 80% of sample reads can be mapped to the pig reference genome, and more than 71.74% reads were unique mapped reads. This result indicates that our sequencing data are suitable for subsequent analyses (Table 1). Based on the criterion of FPKM (Fragments Per Kilobase of transcript per Million fragments mapped) > 2 in at least two out of four samples, a total of 10,735 protein-coding genes were identified as expressed in proliferation and 11,201 genes in differentiation (Figure 2A, Supplementary Table S1). The porcine NOTCH1 gene sequence has not been published in the Ensembl database, and we could not detect this gene when aligned to the porcine reference genome. By using N1ICD sequence BLAST to clean reads, we discovered that NI1CD has 11,704, 314, 206 and 90 reads in P-N1ICD, P-Control, D-N1ICD and D-Control, respectively (Figure 2C). The Notch1 gene sequence published in NCBI database has 14,191, 915, 440 and 306 reads in P-N1ICD, P-Control, D-N1ICD and D-Control, respectively (Figure 2C). This result demonstrates that the expression trend of N1ICD in the gene expression profile is consistent with the result of Figure 1D,E.

Similarly, for miRNA sequencing, we have obtained about 59.74 million raw reads from four samples; after filtering out all low quality reads, about 48.73 million clean reads were identified, and their average Q30 quality scores are about 89% (Table 2). Clean reads were annotated to the Silva database, the GtRNAdb database, the Rfam database and the Repbase database, respectively, to filter out other small RNAs. The result showed that the unannotated clean reads of all the samples are over 71.7%, which is good for the next analysis (Figure 2D). Then, all the unannotated clean reads were analyzed using miRDeep2. After transcripts per million (TPM) were normalized, 385 miRNAs were identified, including 239 known miRNAs and 146 novel miRNAs (Figure 2B and Supplementary Table S2).

### 2.3. Differential Expression of mRNAs in PSCs

Next, based on the criteria of |log_2_FC| > 0.585 (FC: Fold change) and FDR ≤ 0.01 (FDR: False discovery rate), 295 genes were identified as DEGs in the proliferation phase (Supplementary Table S3). There were 181 genes that were |log_2_FC| > 1, and only 29 genes were |log_2_FC| > 2 including 16 upregulated genes and 13 downregulated genes (Table 3). As can be seen from the table, Notch1 downstream genes HES4, HEYL and HEY1 are significantly upregulated; especially HEYL (log_2_FC = 5.38) and HEY1 (log_2_FC = 2.67) are the first and fifth most significant differentially upregulated among 295 DEGs (Supplementary Table S3), respectively. Interestingly, the Notch1 upstream gene JAG1 is also markedly upregulated by 3.48-fold.

**Figure 3:**
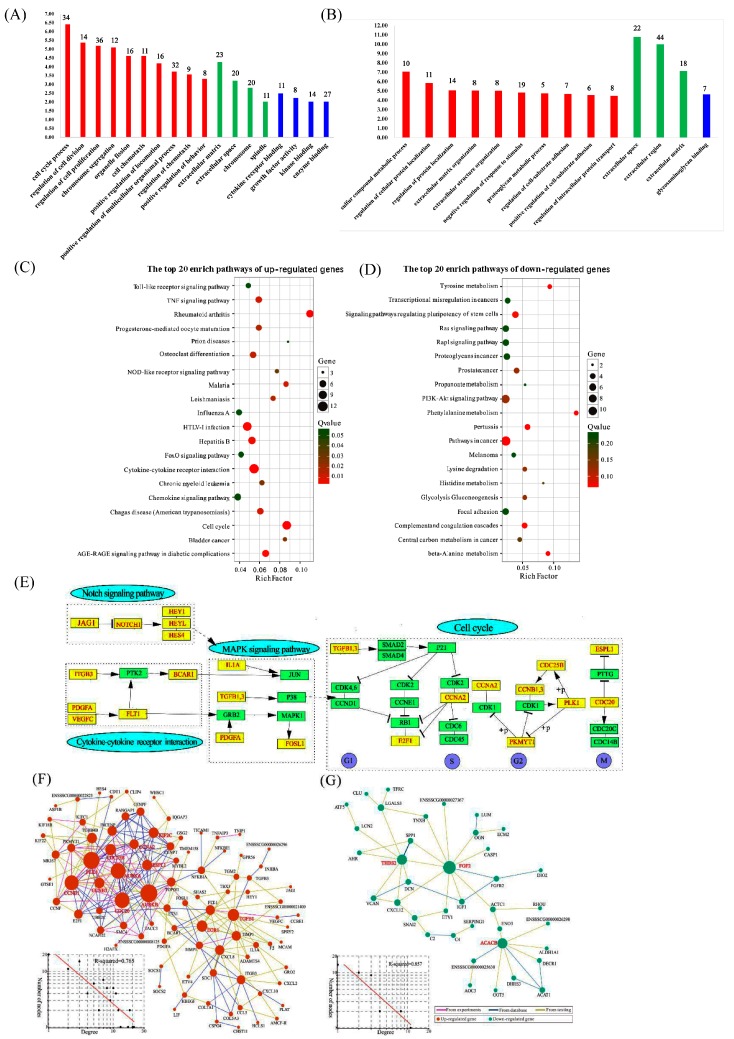
Differential genes functional annotation and interaction in P-N1ICD vs. P-Control. (**A**) Gene Ontology (GO) analysis of upregulated differential genes in proliferation; (**B**) GO analysis of down -regulated DEGs in proliferation; (**C**) Kyoto encyclopedia of genes and genomes (KEGG) analysis of upregulated DEGs in proliferation; (**D**) KEGG analysis of downregulated differential genes in proliferation; (**E**) Up-regulated DEGs in proliferation involve in Notch signaling pathway, Focal adhesion, MAPK signaling pathway and Cell cycle; (**F**) Gene interaction of upregulated DEGs in proliferation; (**G**) Gene interaction of downregulated DEGs in proliferation. Nodes represent genes and edges represent gene-gene interactions. The diameter of each node is proportional to its degree value. This graph was generated using the Cytoscape software. The degree distribution of the network is shown as an inset. The degree distribution follows a power law distribution.

To analyze the function of DEGs, we used GO (Gene Ontology) and KEGG (Kyoto Encyclopedia of Genes and Genomes) for functional annotation. For upregulated enriched genes, there were a total of 18 over-represented terms (Figure 3A, Supplementary Table S4). In the biological process (BP) category, 10 GO terms were significantly over-represented, the top five GO terms namely being cell cycle process, regulation of cell division, regulation of cell proliferation, chromosome segregation and organelle fission, which are associated with cell proliferation. For the cellular component (CC) category, the over-represented GO terms were “extracellular matrix,” “extracellular,” “chromosome” and “spindle”. The over-represented GO terms under the molecular function (MF) category were “cytokine receptor binding,” “growth factor activity,” “kinase binding” and “enzyme binding.” In contrast, for the downregulated enriched gene, a total of 14 GO terms was over-represented (Figure 3B, Supplementary Table S5), including “sulfur compound metabolic process,” “regulation of cellular protein localization,” “regulation of protein localization,” “extracellular matrix organization,” “extracellular structure organization,” “negative regulation of response to stimulus,” “proteoglycan metabolic process,” “positive regulation of cell-substrate adhesion” and “regulation of intracellular protein transport” in the BP category; “extracellular space,” “extracellular region” and “extracellular matrix” in CC category; and “glycosaminoglycan binding” in MF category. Most of GO terms are related to the extracellular matrix (ECM), especially cell adhesion and protein localization. Taken together, upregulated enriched genes are mainly involved in cell proliferation and cell cycle, while downregulated enriched genes are related to cell adhesion and protein localization. KEGG was performed to determine significant pathways involving the upregulated DEGs identified in this study. Our results showed that 43 pathways were significantly enriched for the identified DEGs (*p* < 0.05, Supplementary Table S6); the top 20 of pathway enrichment are shown in Figure 3C. Moreover, “cell cycle,” “cytokine-cytokine receptor interaction,” “osteoclast differentiation,” “chemokine signaling pathway,” “FoxO signaling pathway” and “disease associated pathways” were among the most enriched pathways. Among these enriched pathways, the “cell cycle” pathway is the most significant one, which is consistent with GO analysis. Since the “cell cycle” pathway is the most important, we further examined the relationship between “cell cycle” and other pathways based on KEGG database. To our surprise, both “Notch signaling pathway” and “cytokine-cytokine receptor interaction” (belonging to “focal adhesion”) may indirectly regulate cell cycle through regulating the MAPK signaling pathway (Figure 3E). Based on the same standard, 24 pathways were enriched in downregulated DEGs (Supplementary Table S7). The DEGs were significantly enriched in several pathways associated with protein metabolism; for example, “phenylalanine metabolism,” “tyrosine metabolism,” “beta-alanine metabolism,” “lysine degradation” and “histidine metabolism” (Figure 3D). Other pathways are mainly involved in regulating diseases.

Gene network analysis was performed using integrative gene-gene interaction data from the STRING database (https://string-db.org/) to develop a thorough picture of DEGs at the system level. The reconstructed gene network for upregulated DEGs contains 69 genes and 245 gene-gene interactions (Figure 3F), while the reconstructed gene network for downregulated DEGs consists of 31 genes and 51 gene-gene interactions (Figure 3G). Degree distribution analysis shows that both networks followed a power-law distribution and therefore belonged to scale-free small world networks [2]. Small world networks have the particular feature that some nodes, known as hub genes, are highly connected compared with others. Using “Q-value < 0.01 |log_2_FC| > 0.585” as the threshold value, we identified ten hub genes (aurora kinase B (AURKB), aurora kinase A (AURKA), polo like kinase 1 (PLK1), cell division cycle 20 (CDC20), cyclin B3 (CCNB3), extra spindle pole bodies like 1separase (ESPL1), cyclin A2 (CCNA2), cell division cycle 25B (CDC25B), kinesin family member 2C (KIF2C) and G2/mitotic-specific cyclin-B1 (CCNB1)) involved in the cell cycle process for the network of upregulated DEGs and three hub genes (thrombospondin 2 (THBS2), FGF2 and acetyl-CoA carboxylase beta (ACACB)) for the network of downregulated DEGs. These genes represent functionally important genes due to their key positions in the networks and thus deserve further investigation.

Only nine DEGs were identified during the differentiation of PSCs with overexpressed N1ICD (Table 4). From the table, only ENSSSCG0000000454 (PCNA-associated factor) was |log_2_FC| > 1, which is downregulated.

### 2.4. Differential Expression of Known miRNAs in PSCs

Forty-four and 13 known miRNAs were identified as DEMs, based on the criterion of |log_2_FC| > 0.585 and FDR ≤ 0.01, in the proliferation and differentiation phase, respectively. Among the 44 DEMs, 12 DEMs were upregulated, while 32 DEMs were downregulated when N1ICD was overexpressed (Table 5). By contrast, 11 DEMs were upregulated, and only two DEMs were downregulated in the differentiation phase (Table 6).

Only seven DEMs are over a two-fold change in the proliferation phase of PSCs. They are ssc-miR-935, ssc-miR-145-5p, ssc-miR-146b, ssc-miR-221-3p, ssc-miR-218-5p, ssc-miR-331-5p and ssc-miR-369 (Table 5). To further validate these seven differentially-expressed DEMs identified by the miRNA sequencing, they were subjected to the quantitative stem-loop RT-PCR analysis. In comparison with proliferation, five DEMs are over a two-fold change in the differentiation phase, including ssc-miR-139-3p, ssc-miR-9820-5p, ssc-miR-145-5p, ssc-miR-30b-5p and ssc-miR-324 (Table 6).

### 2.5. Construction of TF-mRNA and TF-miRNA Regulatory Networks

**Figure 4:**
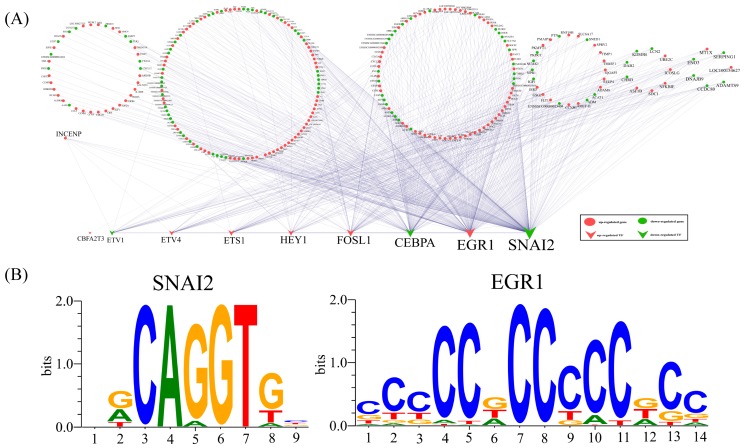

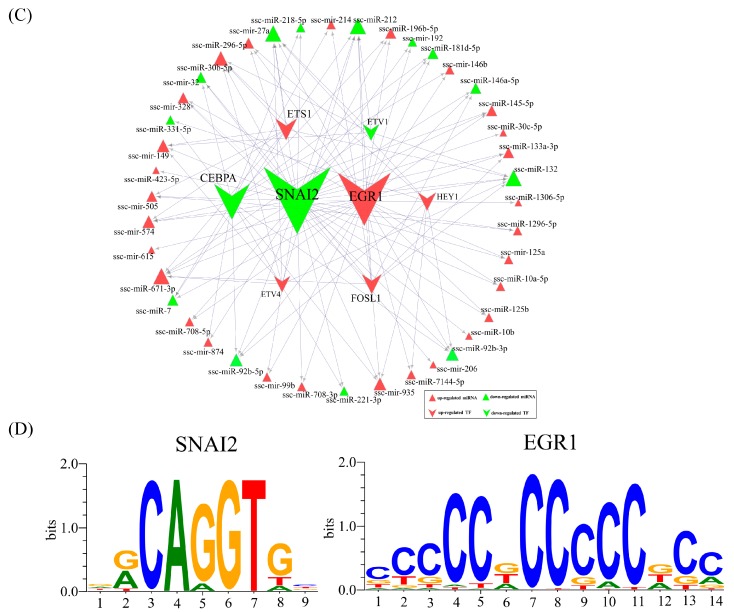
Analyses of TF-mRNA and TF-miRNA networks in P-N1ICD vs. P-Control. (**A**) The network of TF-mRNA in P-N1ICD vs. P-Control; red circular represents upregulated DEGs; green circular represents downregulated DEMs; red V-shape represents upregulated TFs; and green V-shape represents downregulated TFs. (**B**) The sequence logo display for transcription factors of SNAI2 and EGR1 whose binding sites are significantly enriched in the regulation of DEGs in P-N1ICD vs. P-Control. (**C**) The network of TF-miRNA in P-N1ICD vs. P-Control; red triangle represents upregulated DEMs; green triangle represents downregulated DEMs; red V-shape represents upregulated TFs; and green V-shape represents downregulated TFs. (**D**) The sequence logo display for transcription factors of snail family transcriptional repressor 2 (SNAI2) and early growth response 1 (EGR1) whose binding sites are significantly enriched in the regulation of DEMs in P-N1ICD vs. P-Control.

Transcription factors are important in regulating gene expression. We extracted a 2000-bp upstream sequence of the promoter for all DEGs and DEMs according to the principle of promoter prediction of eukaryotic animals [42,43]. Based on the results of TFBSTools software (version 1.16.0) configured with position-weight matrices (PWM) from the JASPAR2016 database, we obtained 338 transcription factors (TFs) regulating the DEGs in P-N1ICD versus P-Control samples, but only nine TFs are differentially expressed, including CCAAT/enhancer binding protein alpha (CEBPA), CAMP responsive element binding protein 3 like 1 (CREB3L1), early growth response 1 (EGR1), ETS proto-oncogene 1 (ETS1), ETS variant 1 (ETV1), ETS variant 4 (ETV4), FOS like 1 (FOSL1), HEY1 and snail family transcriptional repressor 2 (SNAI2). We then used nine differentially-expressed TFs to construct the TF-mRNA and TF-miRNA regulatory networks in P-N1ICD versus P-Control samples, respectively (Figure 4A,B). Both SNAI2 and EGR1 are over-represented hub TFs in TF-mRNA and TF-miRNA regulatory networks, and their binding sites were visualized by WebLogo (Figure 4C,D). Our analysis provides a reference to the regulatory mechanisms underlying the overexpressed N1ICD in PSCs’ proliferation.

### 2.6. Combined Expression Analyses of DEMs and DEGs in PSCs with Overexpressed N1ICD

**Figure 5:**
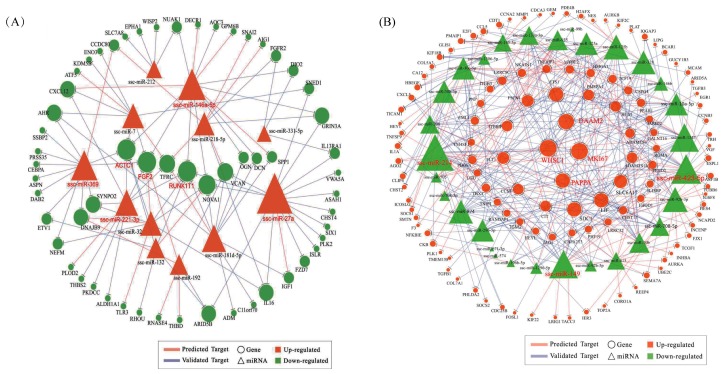

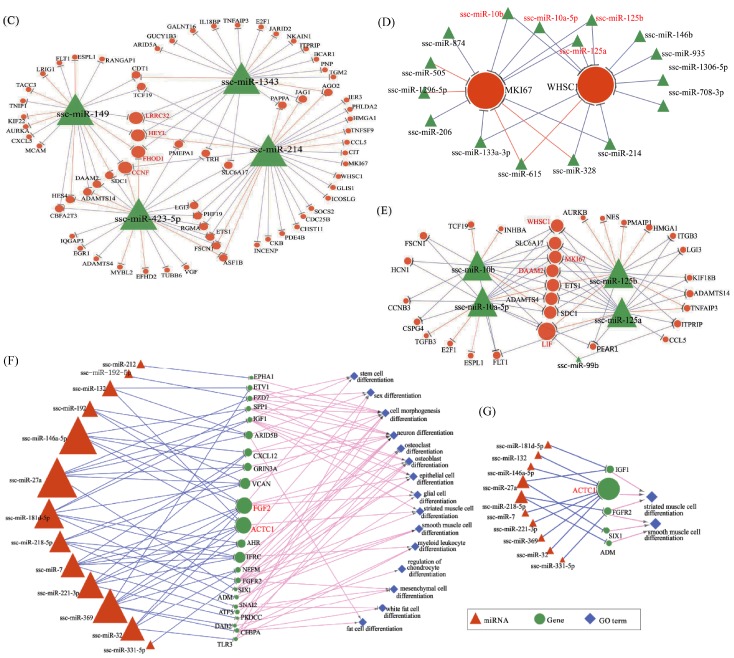
Analyses of mRNA-miRNA networks in P-N1ICD vs. P-Control. (**A**) The network of upregulated miRNAs and downregulated genes in P-N1ICD vs. P-Control. Deep orange triangle represents upregulated miRNA; green circle represents downregulated gene; blue lines represent predicted targeting genes; orange lines represent verification of targeting genes. (**B**) The network of downregulated miRNAs and upregulated genes in proliferation. Green triangle represents downregulated miRNA; deep orange circle represents upregulated gene. (**C**) The network of four hub downregulated miRNAs (ssc-miR-214, ssc-miR-423-5p, ssc-miR-149 and ssc-miR-1343) in P-N1ICD vs. P-Control, the hub genes of which are leucine rich repeat containing 32 (LRRC32), hes related family bHLH transcription factor with YRPW motif-like (HEYL), formin homology 2 domain containing 1 (FHOD1) and cyclin F (CCNF). (**D**) The network of two hub upregulated genes (MKI67 and WHSC1). Both MKI67 and WHSC1 are targeted by ssc-miR-10b, ssc-miR-10a-5p, ssc-miR-125a and ssc-miR-125b, which belong to the miR-10 family. (**E**) The network of five downregulated miRNAs in proliferation, which belong to the miR-10 family. Interleukin 6 family cytokine (LIF) is targeted by all five miRNAs. Three hub genes in the Figure 4B network, including MKI67, WHSC1 and dishevelled associated activator of morphogenesis 2 (DAAM2), are targeted by ssc-miR-10b, ssc-miR-10a-5p, ssc-miR-125a and ssc-miR-125b. (**F**) The network of downregulated miRNAs, upregulated genes and differentiation-related GO terms. Blue rhombus represents differentiation-related GO terms. (**G**) The network of downregulated miRNAs, upregulated genes and muscle cell differentiation-related GO terms. Blue rhombus represents differentiation-related GO terms.

To further study the function of DEMs and DEGs, we focused on the trend of expression changes of DEMs and the target genes. To identify the connection between miRNAs and mRNAs, we used miRwalk 2.0 software to predict their target genes. We considered only those genes with the expression pattern that is negatively correlated with its targeting miRNAs. After combining the DEGs and target genes, we obtained 338 and 94 miRNA-mRNA interaction pairs in downregulated miRNAs and upregulated miRNAs in proliferating PSCs, respectively. Furthermore, we used Cytoscape software to visualize and analyze miRNA-mRNA networks (Figure 5A,B). GO and KEGG analyses were used for all target DEMs to annotate their functions. GO analysis on predicted upregulated target DEGs of downregulated DEMs revealed that biological processes associated with cell proliferation, cell cycle, cell division, mitosis, response to lipid and organelle fission were among the highly enriched GO terms (Supplementary Table S8). Apart from this, 39 significant KEGG pathways were enriched by the predicted upregulated DEGs. Besides the pathways related to disease, pathways associated with cytokine-cytokine receptor interaction, cell cycle, osteoclast differentiation, focal adhesion, ECM-receptor interaction and progesterone-mediated oocyte maturation were more enriched (Supplementary Table S9). Cell cycle regulation appeared in the result of both GO and KEGG analyses, which remains consistent with the result of Figure 3A,C. There were 40 significant biological processes GO terms enriched by targeting downregulated DEGs (Supplementary Table S10). Additionally, 14 KEGG pathways were enriched by the predicted downregulated DEGs (Supplementary Table S11).

To further study four hub miRNAs (ssc-miR-214, ssc-miR-423-5p, ssc-miR-149 and ssc-miR-1343) in Figure 4B, we constructed a miRNA-mRNA network alone. HEYL, cyclin F (CCNF), formin homology 2 domain containing 1 (FHOD1) and leucine rich repeat containing 32 (LRRC32) as the hub genes are targeted by three miRNAs (Figure 5C). Similarly, both of the hub genes (WHSC1 and MKI67) are targeted by the miR-10 family, including ssc-miR-10, ssc-miR-10a-5p, ssc-miR-125a and ssc-miR-125b (Figure 5D). Interleukin 6 family cytokine (LIF) is targeted by all five miR-10 families (Figure 5E). In contrast to upregulated DEGs, the biological processes of downregulated DEGs associated with cell adhesion, regulation of cell differentiation and cell migration were more important in proliferating PSCs with overexpressed N1ICD. There are 23 downregulated DEGs associated with differentiation, including fibroblast growth factor 2 (FGF2) and actin, alpha, cardiac muscle 1 (ACTC1) two hub genes (Figure 5F). Five genes (ACTC1, insulin like growth factor 1 (IGF1), adrenomedullin (ADM), SIX homeobox 1 (SIX1) and fibroblast growth factor receptor 2 precursor (FGFR2)) are related to muscle cell differentiation (Figure 5G). These results suggest that most of the downregulated DEGs are mainly related to differentiation, migration and adhesion. Since the number of targeting downregulated genes is too small, it is meaningless to identify pathways by the KEGG database.

### 2.7. Quantitative RT-PCR Validation

**Figure 6:**
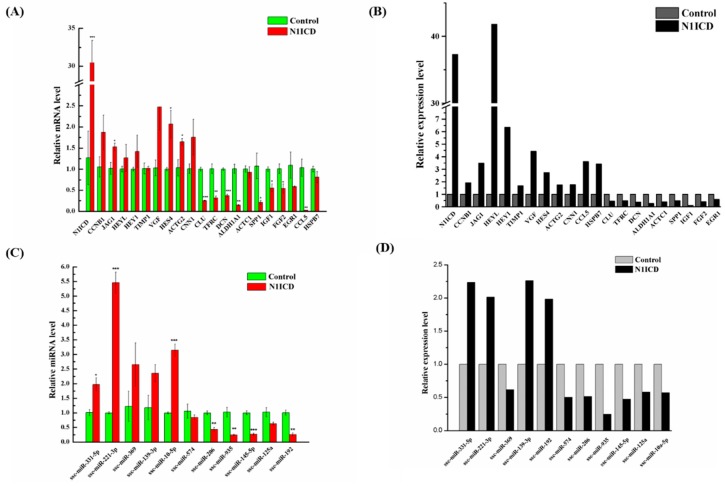
Quantitative RT-PCR validation. (**A**) Quantitative RT-PCR validated DEGs. DEGs (N1ICD, G2/mitotic-specific cyclin-B1 (CCNB1), Jagged 1 (JAG1), HEYL, HEY1, TIMP metallopeptidase inhibitor 1 (TIMP1), VGF nerve growth factor inducible (VGF), HES4, clusterin (CLU), transferrin receptor (TFRC), decorin (DCN), aldehyde dehydrogenase 1 family member A1 (ALDH1A1), actin, alpha, cardiac muscle 1 (ACTC1), secreted phosphoprotein 1 (SPP1), fibroblast growth factor 2 (FGF2) and C-C motif chemokine ligand 5 (CCL5)) belong to P-N1ICD vs. P-Control, and DEGs (actin, gamma 2, smooth muscle, enteric (ACTG2), calponin 1 (CNN1), heat shock protein beta-7 (HSPB7) and EGR1) belong to D-N1ICD vs. D-Control. (**B**) DEGs in gene expression profile. (**C**) Quantitative RT-PCR validated DEMs. DEMs (ssc-miR-221-3p, ssc-miR-369, ssc-miR-10-5p, ssc-miR-206, ssc-miR-935, ssc-miR-145-5p, ssc-miR-125a and ssc-miR-192) belong to P-N1ICD vs. P-Control, and DEMs (ssc-miR-331-5p, ssc-miR-139-5p and ssc-miR-574) belong to D-N1ICD vs. D-Control. (**D**) DEMs in the gene expression profile.

We used qRT-PCR to validate the gene expression profile and small RNA data using 17 DEGs in P-N1ICD versus P-Control and four DEGs in D-N1ICD versus D-Control (Figure 6A; Supplementary Table S12–S14). The corresponding result of gene expression profiling is presented in Figure 6B. Similarly, eight DEMs in P-N1ICD versus P-Control and three DEMs in D-N1ICD versus D-Control were used to validate by qRT-PCR (Figure 6C). The corresponding result of small RNA sequencing is presented in Figure 6D. Although there was different fold changes between sequencing data and qRT-PCR, the expression patterns were consistent between these two methods. Thus, the data of the gene expression profile and small RNAs have a high quality, which is credible.

## 3. Discussion

Our study, we believe, is the first comprehensive report about the miRNA and mRNA signatures in PSCs with overexpressed NI1CD, and the overall findings of this study will facilitate our understanding of the molecular events underlying the roles of microRNAs and genes in the Notch signaling pathway in regulating PSCs’ development.

Notch signaling has been identified to be critical in modulating satellite cell function, and it can be used as a therapeutic application to overcome skeletal muscle atrophy [39,44]. However, all previous research used only methods of transient transfection of NICD, which usually has the problems of low transfection efficiency and short duration. In our study, we constructed a stably-transfected N1ICD model to avoid the problems mentioned above. In Figure 1A, Notch1 was mainly located in the nucleus of PSCs. In Figure 1B, the green fluorescence in the pEGFP-N1ICD group was higher than the control group, which suggests that N1ICD overexpression was successful in PSCs. However, the degree of green fluorescence of the pEGFP-N1ICD group was lower. A possible explanation is the transfecting N1ICD fragment changed the skeleton of the vector, which affects the ability of the protein expression of the vector.

In 2016, Shan et al. reviewed that activation of Notch1 signaling promotes the muscle satellite cells’ quiescence and proliferation [45]. To date, several studies have confirmed several specific genes and miRNAs regulating the proliferation of satellite cells through the Notch signaling pathway [39,40,46]. However, the regulated network of the Notch signaling pathway has still not been explored in proliferating satellite cells. In this study, our Edu experiment indicated that overexpression of N1ICD promotes PSCs’ proliferation. Meanwhile, the Q-PCR result showed that PAX7 dramatically increases while P21, also known as cyclin-dependent kinase inhibitor 1, significantly decreases PSCs proliferation with overexpressed N1ICD. To further uncover the regulating network in PSCs proliferation, we used NGST to assess the expression change in both miRNAs and mRNAs. A total of 349 miRNAs and 10,735 genes was identified in both proliferation and differentiation of PSCs. Furthermore, 44 miRNAs and 295 genes were significantly different in satellite cell proliferation by using integrative methods. After analyzing the sequencing results using NGST, we found the porcine N1ICD was approximately 1.6-fold higher after N1ICD overexpression. The Notch downstream regulated genes HEYL (about 41.6-fold), HEY1 (about 6.36-fold) and HES4 (about 2.73-fold) were also upregulated during PSCs proliferation. HEYL is the most prominent gene among all DEGs that were significantly changed. Interestingly, JAG1 (about 3.48-fold) was also increased. Moreover, HEYL, HEY1 and JAG1 are known for regulating skeletal muscle development [47]. However, this was not so for HES4, which implies that HES4 may be another gene involved in regulating skeletal muscle development. Two myomiRs, miR-133a-3p (about 1.78-fold) and miR-206 (about 1.94-fold), which are known to increase skeletal muscle differentiation, were downregulated [48,49,50,51]. Indeed, overexpressed N1ICD promotes the Notch signaling pathway and downregulates myomiRs relating to differentiation, which implies promoting PSCs’ proliferation.

To investigate in depth how PSCs proliferation changes in the gene alteration of overexpressed N1ICD, we took advantage of bioinformatics analysis. The GO and KEGG analyses provided substantial evidence that upregulated DEGs are mainly associated with cell cycle regulation, because “cell cycle” was the most enriched in both GO and KEGG analyses. The STRING database network also declares upregulated DEGs mostly related to cell cycle. Numerous genes, including CCNA2, CCNB1, CCNB3, CDC20, CDC25B, E2F transcription factor 1 (E2F1), ESPL1, protein kinase, membrane associated tyrosine/threonine 1 (PKMYT1), PLK1, transforming growth factor beta 1 (TGFB1) and transforming growth factor beta 3 (TGFB3), are enriched in the “cell cycle” pathway. CCNA2, CCNB1, and CCNB3 belong to the cyclin family, whose members regulate cell cycle progression by interacting with CDK kinases [52]. Notch signaling has been reported to be involved in regulating the cell cycle process. Abnormal high Notch signaling in T-cell acute lymphoblastic leukemia (T-ALL) keeps S-phase kinase associated protein 2 (SKP2) at a high level and lowers p27Kip1, leading to more rapid cell cycle progression [53]. In adult muscle, Notch inhibition by gamma-secretase inhibitor (GSI) caused prompt upregulation of cyclin-dependent kinase (CDK) inhibitors (p15, p16, p21 and p27), whereas activation of Notch inhibits them [54]. Our laboratory has demonstrated that knocking down Notch1 reduced the expression of cell cycle-related genes (cyclin B1, D1, D2 and E1), which decreased the proliferation of PSCs [18]. Similarly, CDC20 and CDC25B as cell-division cycle proteins are also essential to regulate the cell cycle process. The most important function of CDC20 is to activate the anaphase-promoting complex (APC/C). APC/C is a large complex containing 11–13 subunits, which initiates chromatid separation and entrance into anaphase [55,56]. CDC25B encodes the enzyme M-phase inducer phosphatase 2 in humans [57]. All above analyses indicate that the activation of the Notch signaling pathway in PSCs mainly promotes cell proliferation through regulating the cell cycle.

We used the KEGG database to further unveil how the Notch signaling pathway regulates cell cycle. Since the MAPK signaling pathway has been identified to regulate the cell cycle directly in the KEGG database, we suspect that the Notch signaling pathway may regulate the MAPK signaling pathway. Besides, a great many research works demonstrated that there is a close relationship between the Notch signaling pathway and the MAPK signaling pathway based on the result of KEGG analysis. Angiotensin II activates MAPK signaling in ductus arteriosus smooth muscle cells, which was counteracted by gamma-secretase inhibitor DAPT, a Notch signaling inhibitor [58]. Furthermore, Notch3 protects vascular smooth muscle cell (VSMC) apoptosis by upregulating three pro-survival genes, which is mediated through MAPK signaling [59]. However, no one has studied if the Notch signaling pathway regulates skeletal satellite cells through mediating the MAPK signaling pathway. Based on the above data, in Figure 3E, we delineated that the Notch signaling pathway indirectly regulates the cell cycle in PSCs though mediating the MAPK signaling pathway.

To study the regulation of DEGs and DEMs, the TF-mRNA and TF-miRNA networks in P-N1ICD versus P-Control were constructed. Interestingly, SNAI2 and EGR1 as the transcription factors are hub TFs in both the TF-mRNA and TF-miRNA network.

SNAI2 belongs to the Snail gene family, which encodes DNA-binding zinc finger proteins that function as transcription repressors in mammals [60]. In mice muscle, SNAI1/2 recruit HDAC1/2 (as co-repressor inhibiting CSL in the Notch signaling pathway, which was annotated in the KEGG database) to block myoblast entry into differentiation [61]. In our data, SNAI2 is also a DEG in P-N1ICD versus P-Control, so the downregulation of SNAI2 may promote PSCs’ proliferation and entry into differentiation. EGR1 is also a zinc-finger transcription factor, which plays a pivotal role in muscle proliferation [62,63]. In addition, EGR1 binds to the osteopontin (OPN) promoter and upregulates OPN expression, which promotes vascular smooth muscle cells’ (VSMCs) proliferation [64]. In our data, the EGR1 upregulation may contribute to PSCs’ proliferation.

To investigate how miRNAs exactly participate in the gene alteration of overexpressed N1ICD, we constructed miRNA-mRNA networks. Through the integration of miRNA and mRNA expression data and miRNA-mRNA target prediction analysis, many putative miRNA-mRNA interactions were identified. Highly connected nodes in biological networks are known as hubs. Hubs are topologically important to the structure of the network; they are also known to be preferentially associated with various phenotypes of interest [65]. The relative importance of a hub node, however, can change depending on the biological context. Since hub nodes are known to play important roles in many networks, we also looked for the presence of hub miRNAs. Several downregulated hub miRNAs were identified, including ssc-miR-214, ssc-miR-423-5p, ssc-miR-149 and ssc-miR-1343. Additionally, each of these hub miRNAs targets more than 21 genes. Interestingly, all these hub miRNAs target the HEYL gene. miR-214 mediated Ezh2 protein reduction, which accelerates skeletal muscle cell differentiation [34]. Ectopic expression of miR-149 in gastric cancer cells inhibits proliferation and cell cycle progression by downregulating zinc finger and BTB domain containing 2 (ZBTB2) [66]. Though ssc-miR-125a, ssc-miR-125b, ssc-miR-10a-5p, ssc-miR-10b, ssc-miR-935 and ssc-miR-145 are not the hub miRNAs, each of them also targets more than 14 genes and associated with inhibiting proliferation. In our research, miR-125a, miR-125b, miR-10a-5p and miR-10b belong to the miR-10 family, which are most abundant among all significant differential miRNAs and target two hub genes (WHSC1 and MKI67). miR-10 members play key roles in the differentiation process of human mesenchymal stem cells [67], neuroblastoma [68] and smooth muscle cells [69]. Inhibition of miR-10a in smooth muscle cells abrogates their retinoic acid-induced differentiation, likely upregulating HDAC4, an identified target gene of miR-10a [69]. However, miR-10a alone was unable to drive the differentiation of smooth muscle cells [69]. miR-125a serves as a tumor suppressor has been shown to inhibit proliferation in different cancers [70,71,72,73]. Overexpressing miR-125a-5p inhibits C2C12 myoblast proliferation by targeting E2F3 [74]. miR-125b has also been found to suppress proliferation in different kinds of cancer [75,76,77]. miR-935 suppresses tumorigenesis of gastric signet ring cell carcinoma by targeting Notch1 [78]. miRNA-145 inhibits VSMC proliferation by targeting CD40 [79]. VSMC differentiation signals potentially can be fine-tuned or amplified by miR-143/145 through Notch and SRF in parallel pathways [80]. Similarly, two upregulated hub genes were identified including MKI67 and WHSC1. MKI67 protein (also known as Ki-67) is a cellular marker for proliferation, which appears during the cell cycle (G1, S, G2 and mitosis), but is absent from the resting cells phases (G0) [81]. In a previous study, WHSC1, a cell-cycle checking point protein, was shown to play important roles in human carcinogenesis [82]. By contrast, upregulated hub miRNAs include miR-27a, miR-146a-5p, miR-369, miR-181d-5p and miR-221-3p; and each of these hub miRNAs targets more than nine genes. miR-27a depressed the expression of myostatin and promoted porcine myoblast proliferation [25,26]. miR-146a promotes C2C12 proliferation by inhibiting the Numb gene, which promotes satellite cell differentiation by inhibiting Notch signaling [40]. Ectopic expression of miR-221, as well as miR-222 in differentiating myoblasts delayed their withdrawal from the cell cycle and subsequent myogenesis through P27, their target gene [28]. Meanwhile, two downregulated genes were identified as hub genes, including RUNX1T1 and FGF2. In murine embryonic stem cells, Runx1t1 expression is upregulated during hematopoietic differentiation [83]. Furthermore, a low level of Runx1t1 expression was detected in undifferentiated human ESCs (embryonic stem cells), and a gradual increase in expression was observed during differentiation [84]. All these data seem to indicate that RUNX1T1 plays a role during stem cell differentiation, at least in the hematopoietic, neuronal and muscular cells. In 2016, Shelton et al. found that collagenase IV and continued FGF2 supplementation expanded differentiating skeletal muscle progenitors about three-fold every two weeks. All above-mentioned hub genes and miRNAs may regulate the proliferation of N1ICD overexpressed PSCs.

Presently, much research has demonstrated that the Notch signaling pathway also regulates the differentiation of skeletal satellite cells. In C2C12 cells, activation of the Notch signaling pathway significantly inhibits myogenic differentiation and differentiation-associated genes [85,86,87]. Furthermore, in mice primary myoblasts, myogenic cell lineage-specific overexpressed N1ICD decreases the expression of MYOD, and MYOG and inhibits myogenic differentiation [88]. Besides, inhibition of Notch signaling would be suitable for a rapid temporal switch to activate Wnt signaling, which facilitated satellite cells’ differentiation [20]. By contrast, overexpressed N1ICD dramatically reduce the expression of MYHC, MYOD and MYOG, which implies the inhibition of PSCs’ differentiation. However, in our sequencing data, only a few genes appeared significantly differential in the differentiation of satellite cells, including EGR1, ACTG2 and CNN1. Knock-down of EGR1 inhibited differentiation of bovine skeletal muscle-derived satellite cells. Therefore, overexpressed N1ICD in PSCs differentiation downregulated the expression of EGR1, which may inhibit PSCs’ differentiation [89]. However, ACTG2 and CNN1 are not known to be associated with satellite cell differentiation.

Although our study has identified the differentially-expressed genes, miRNAs and the interaction network in NICD1 overexpressed PSCs, the molecular mechanisms of how these genes, miRNAs and related TFs regulate PSCs development need to be further studied.

## 4. Materials and Methods

### 4.1. Ethics Statement

All animal procedures were conducted according to the guidelines developed by the China Council on Animal Care, and experimental protocols had received the approval of the Animal Care and Use Committee of Guangdong Province, China (2017A109, 5 August 2017).

### 4.2. Construction of a N1ICD Overexpressing Cell Line

The experimental procedure of isolation and culture for primary PSCs has been described in detail previously [18]. N1ICD was cloned from PSCs cDNA. The coding region was subcloned into a mammalian expression vector, pEGFP-N1. To get the constitutively N1ICD-expressing cell, pEGFP-N1ICD was transfected into PSCs; as a negative control, cells were transfected with pEGFP-N1. G418 was added 24 h later to select the stable cell clones. Positive cells were induced to proliferation at Day 1 and differentiation at Day 7, respectively. Additional positive cells were immediately snap-frozen in liquid nitrogen and stored at −80 °C until use.

### 4.3. Immunocytochemistry and Edu Assay

PSCs were washed with PBS three times and fixed in paraformaldehyde. Then, cells were permeabilized with 1% TritonX-100 in PBS for 10 min at room temperature. After that, cells were incubated in 5% bovine serum albumin (BSA), primary antibodies (N1ICD (#3608), Cell Signaling Technology, Beverly, MA, USA; Myosin Heavy Chain Antibody(#MAB4470), R&D Systems, Minneapolis, MN, USA) and secondary antibodies (Goat anti-Rabbit IgG (H + L)-HRP(#BS13278), Bioworld Technology, Suite 500, St. Louis Park, MN, USA; Goat anti-Mouse IgG (H + L)-HRP(#BS12478), Bioworld Technology, Suite 500 St. Louis Park, MN, USA), successively. Finally, DAPI was added to stain the nuclei. Photographs were taken for the future analysis.

PSCs were seeded in 48-well plates (1 × 10^5^ cells per well). PSCs were transfected with pEGFP-N1ICD and the control by Lipofectamine 2000 (Invitrogen, Carlsbad, CA, USA), according to the manufacturer’s instructions. After 6 h, the medium was replaced with a new growth medium. After having been transfected for 24 h, these PSCs were incubated with Edu solution containing growth medium (RiboBio, Guangzhou, China) for 2 h. Subsequently, these cells were washed with PBS and fixed with 80% acetone. The fixed PSCs were incubated with 0.5% TritonX-100 and washed with methyl alcohol and PBS, respectively. Then, PSCs were incubated with 1×Apollo (RiboBio, Guangzhou, China) for 30 min. Then, PSCs were incubated with 0.5% TritonX-100 (Sigma-Aldrich, St. Louis, MO, USA) for 10 min and DAPI (Lot. 10A08A77; BOSTER Biological Technology C0., Lid, Wuhan, China) for 10 min, respectively. Last, PSCs were washed with PBS and observed with a Nikon TE2000-U inverted microscope (Nikon Instruments, Tokyo, Japan), and images of the proliferating PSCs were captured with NIS-Elements BR Basic Research software (version 4.60.00, Nikon Corporation, Tokyo, Japan). Counting proliferating Edu positive PSCs was done using ImagePro Plus (Media Cybernetics, Inc., Silver Spring, MD, USA).

### 4.4. RNA Extraction and Sequencing

The method used for the total RNA extraction from PSCs has been described in detail previously [18]. RNA purity was checked using the NanoDrop ND-2000 spectrophotometer (Thermo Scientific, Waltham, CA, USA), and the average A260/280 and A260/230 ratios were 1.87 and 1.70, respectively. RNA integrity was assessed using the RNA Nano 6000 Assay Kit of the Agilent Bioanalyzer 2100 system (Agilent Technologies, Santa Clara, CA, USA). Only the RNA samples with an RNA integrity number (RIN) ≥7 were used for subsequent experiments. After library preparation, all the samples were used for 50 base-pair single-end mRNA and miRNA sequencing on the Illumina Hiseq 2500 platform.

### 4.5. Analysis of mRNA Sequencing Data

The in-house perl scripts in the fastq format were first used to process the raw data (raw reads). Any raw data (raw reads) containing adapter, ploy-N or low quality were removed to obtain the clean data (clean read). The quality of raw data was also checked by FastQC software. All the downstream analyses were based on high-quality clean data. The clean reads were then mapped to the pig reference genome [90] using Tophat2 with one mismatch tolerance. Once the sample reads were aligned to the pig genome, the expression of genes for each sample was normalized to fragments per kilobase of the exon model per million fragments mapped {FPKM, FPKM = cDNA Fragments/[Mapped Reads (Millions) × Transcript Length(kb)]}. Some porcine genes, which have not been published in Ensembl database, cannot be aligned to the porcine reference genome. Thus, to assess the expression of these unpublished genes, their sequences were used to align to the clean reads by the Basic Local Alignment Search Tool (Blast 2.4.0+) using parameters of the two-base mismatch and E value < 1 × 10^−5^. For each sequenced library, before differential gene expression analysis, the read counts were adjusted by the edgeR program package through one scaling normalized factor. Differential expression analysis of two samples was performed using the DEGseq (2010) R package. The *p*-value was adjusted using the q-value [91]. q-value < 0.01 |log_2_FC| > 0.585 was set as the threshold for significant differential expression.

### 4.6. Analysis of miRNA Sequencing Data

Raw reads were first checked by fastQC software (version 0.11.5) and processed through in-house perl scripts. In this step, clean reads were obtained by removing reads containing adapter, reads containing poly-N and low quality reads from raw reads. The reads were trimmed and cleaned by removing the sequences smaller than 18 nt or longer than 30 nt. All the downstream analyses were based on the high-quality clean reads. By using Bowtie, the clean reads were mapped to the Silva database, the GtRNAdb database, the Rfam database and the Repbase database for ribosomal RNA (rRNA), transfer RNA (tRNA), small nucleolar RNA (snoRNA) and repeat sequences, respectively. The remaining reads were used to detect known miRNA and predict novel miRNA by miRDeep2 software [92]. The expression profile of sRNA was normalized in transcripts per million [TPM, TPM = reads (sRNA)/total reads × 10^6^]. Differential expression analysis of miRNAs was conducted by using the DEGseq of R package. The *p*-value was adjusted using the q-value [91]. q-value < 0.01 |log_2_FC| > 0.585 was set as the criterion for significant differential expression.

### 4.7. GO, KEGG and STRING Network Analyses

GO and KEGG enrichment analyses of the DEGs were conducted using WebGestalt [93]. The Benjamini–Hochberg procedure was applied to control the false discovery rate (*p* < 0.05). GO analysis was performed to infer the functional consequences of their regional expression pattern. Over-represented GO terms are allocated to three categories: biological process (BP), cellular component (CC) and molecular function (MF). The KEGG database was used to analyze genes involved in different pathways. The DEGs network was generated using the STRING database v10.0 with the criterion of medium confidence (0.400) [94].

### 4.8. Prediction of Transcription Factor Binding Sites and Construction of TF-mRNA and TF-miRNA Regulatory Networks

The transcription factors (TF) of DEGs and DEMs were predicted by the TFBSTools (an R language package for transcription factor binding site analysis [95]) [96], which provides functions to query and manipulate the JASPAR2016 database efficiently. The DNA sequences of these genes and pre-miRNAs upstream of the transcription start site were downloaded from the Ensembl database, including the 2000-bp upstream region. Because of no available pig TF in the JASPAR database (http://jaspardev.genereg.net/), we use the human TF to replace. The relative profile score threshold was set at 95% as a strict screening criterion. Based on the above results, differentially-expressed TFs were screened out to construct TF-mRNA and TF-miRNA regulatory networks, which were visualized by Cytoscape v3.30 software [97]. The sequence logos of motifs of hub TFs in the networks were visualized by WebLogo 3 software (http://weblogo.threeplusone.com/#) [98].

### 4.9. Prediction of miRNA Targets and Construction of miRNA-mRNA Regulatory Networks

Our strategy for identifying miRNA-mRNA regulatory relationships was based on two criteria: computational targets’ prediction and negative regulation relationship. Putative targeting genes of DEMs were predicted by six bioinformatic algorithms (miRwalk, DIANAmT, miRanda, RNA22, RNAhybrid and Targetscan) using the online software miRwalk 2.0 [99]; only the targeting records identified by more than four algorithms or confirmed by the experimental data were used as our selection criterion. Because of the absence of porcine miRNAs in the current version of the abovementioned algorithm, predictions were performed using human miRNAs. Only conserved miRNAs and conserved target genes were included. The Cytoscape software was applied for visualization and analysis of the miRNA-mRNA network. A regulatory effect of less than −0.3 was considered to represent a negative regulatory relationship.

### 4.10. Quantitative Real Time-PCR of DEGs and DEMs

Relative expression levels of the DEGs and DEMs were quantified by real-time PCR. Primer sequences and PCR conditions are listed in Supplementary Table S1. The relative expression of mRNAs and microRNAs levels was normalized with GAPDH and U6 levels, respectively. The experimental procedure of real-time PCR has been described in detail previously [18].

### 4.11. Statistical Analyses

All data are expressed as the mean ± standard error of the mean (S.E.M.) after Student’s *t*-test analysis. The statistical analyses were performed using the R programming tool. A *p*-value < 0.05 was considered statistical significant.

## Figures and Tables

**Table 1:** Gene expression profile data statistics. PRO, proliferation; DIF, differentiation.

Samples	ID	Clean Reads	Unique Mapped Reads %	GC Content %	Q30 %
PRO N1ICD	E1	14,210,217	71.74	56.63	85.81
PRO Control	E2	12,906,715	72.37	55.5	86.16
DIF N1ICD	E3	14,132,305	73.29	56.53	86.38
DIF Control	E4	13,861,909	74.14	54.73	86.81

Samples: PRO N1ICD (E1) represents N1ICD overexpression in proliferation. PRO Control (E2) represents control in proliferation. DIF N1ICD (E3) represents N1ICD overexpression in differentiation. DIF Control (E4) represents control in differentiation. ID represents sample serial numbers. Clean reads were done after removing impurity reads for raw reads. Unique mapped reads are the percentage of each sample aligned to pig reference genome. GC Content is the G and C base content. Q30 % is the percentage of sequencing quality.

**Table 2:** miRNA expression profile data statistics.

Samples	ID	Raw Reads	Containing ‘N’ Reads	<18 nt Reads	>30 nt Reads	Clean Reads	Q30 (%)
PRO N1ICD	E1	13,298,310	2574	801,735	1,426,700	11,067,301	91.62
PRO Control	E2	16,347,926	3066	844,391	2,185,614	13,314,855	88.56
DIF N1ICD	E3	15,553,685	2873	775,545	1,839,473	12,935,794	89.98
DIF Control	E4	14,542,636	2826	1,111,601	2,011,396	11,416,813	87.69

ID: sample serial numbers. Raw reads: raw reads numbers. Containing ‘N’ reads: the reads contain N bases. <18 nt reads: all reads are less than 18 nucleotides. >30 nt reads: all reads are more than 30 nucleotides.

**Table 3:** Differential gene screening in proliferation.

Ensembl_ID	Gene	E1	E2	FDR	log_2_FC
ENSSSCG00000024651	HEYL	2.84	0.07	0	5.38
ENSSSCG00000021865	INHBA	29.63	2.4	0	3.62
ENSSSCG00000011808	SST	16.95	1.44	1.36E-09	3.56
ENSSSCG00000000647	OLR1	2.97	0.43	0.0383193	2.79
ENSSSCG00000006159	HEY1	2.89	0.46	4.16E-07	2.67
ENSSSCG00000009320	FLT1	3.26	0.52	1.46E-09	2.66
ENSSSCG00000008090	IL1A	17.03	3.28	4.60E-11	2.37
ENSSSCG00000011596	TRH	9.59	1.91	1.44E-05	2.33
ENSSSCG00000008954		20.29	4.46	0.0472039	2.19
ENSSSCG00000000910	SOCS2	19.29	4.27	0	2.18
ENSSSCG00000027315		3.63	0.81	8.74E-06	2.17
ENSSSCG00000022576	VGF	512.65	115.68	4.44E-16	2.15
ENSSSCG00000008953	CXCL8	64.99	14.87	5.55E-16	2.13
ENSSSCG00000023165	SEMA7A	18.7	4.32	0	2.11
ENSSSCG00000029691	CORO1A	5.27	1.25	6.67E-06	2.08
ENSSSCG00000002501	BDKRB1	7.1	1.69	0.0001321	2.07
ENSSSCG00000030575	TMEM158	19.08	4.69	6.99E-12	2.03
ENSSSCG00000000246		223.16	55.41	0	2.01
ENSSSCG00000027543		1.75	25.11	0.0637557	−3.84
ENSSSCG00000003439	DHRS3	1.1	13.71	0	−3.63
ENSSSCG00000011700	CP	0.2	2.04	0.024046	−3.36
ENSSSCG00000000857	IGF1	1.45	13.76	1.69E-08	−3.25
ENSSSCG00000023909	THPO	0.28	2.5	0.0750879	−3.17
ENSSSCG00000008397	EFEMP1	2.61	15.43	0	−2.57
ENSSSCG00000007379	WISP2	1.4	7.64	1.09E-08	−2.45
ENSSSCG00000027367		2.41	13.07	0.0066149	−2.44
ENSSSCG00000024122	CHST8	1.23	6.58	1.12E-05	−2.42
ENSSSCG00000016437	WDR86	0.6	3.22	0.0123033	−2.42
ENSSSCG00000004452	PRSS35	1.41	7.32	2.13E-06	−2.38
ENSSSCG00000027724		0.99	4.91	0.0001417	−2.32
ENSSSCG00000001427	C4A	4.09	18.51	0	−2.18
ENSSSCG00000001787	IL16	0.5	2.2	0.0005763	−2.13
ENSSSCG00000010414	CXCL12	20.46	89.14	0	−2.12
ENSSSCG00000016381	SNED1	0.9	3.71	2.55E-10	−2.04
ENSSSCG00000017383	AOC3	0.68	2.76	0.002278	−2.02

FC: Fold change; FDR: False discovery rate.

**Table 4:** Differential gene screening in differentiation.

Ensembl_ID	Gene	E3	E4	FDR	log_2_FC
ENSSSCG00000023899	LOC102158881	6.9	3.79	9.77E-03	0.87
ENSSSCG00000013614	CNN1	25.09	14.2	1.06E-03	0.82
ENSSSCG00000026236		32.41	19.97	7.60E-03	0.7
ENSSSCG00000003517		171.11	96.91	2.34E-03	0.82
ENSSSCG00000008294	ACTG2	126.85	72.21	1.50E-04	0.81
ENSSSCG00000026732		33.94	22.22	4.69E-03	0.61
ENSSSCG00000011643		31.15	19.88	6.95E-03	0.64
ENSSSCG00000014336	EGR1	27.49	46.31	3.60E-04	−0.75
ENSSSCG00000004554		13.37	23.78	2.89E-03	−1.06

**Table 5:** Differential miRNA screening in P-N1ICD vs. P-Control.

miRNA	E1	E2	FDR	log_2_FC
ssc-miR-132	46.8	23.8	0	0.98
ssc-miR-146a-5p	83.1	50.9	0	0.71
ssc-miR-181d-5p	14.7	8.3	0.00113	0.83
ssc-miR-192	7.1	3.6	0.00885	0.99
ssc-miR-212	271	138	0	0.97
ssc-miR-218-5p	5.7	2.6	0.00888	1.15
ssc-miR-221-3p	6087.7	3024.5	0	1.01
ssc-miR-27a	2572.6	1475.6	0	0.8
ssc-miR-32	24.5	15.6	0.00057	0.65
ssc-miR-331-5p	6.9	3.1	0.00427	1.16
ssc-miR-369	3.8	1.3	0.00904	1.56
ssc-miR-7	8936.1	5785.7	0	0.63
ssc-miR-10a-5p	1152.1	2018.5	0	−0.81
ssc-miR-10b	10,288.3	16743	0	−0.7
ssc-miR-125a	487.2	838.8	0	−0.78
ssc-miR-125b	4419.7	6635.1	0	−0.59
ssc-miR-1296-5p	20.6	36.5	0	−0.82
ssc-miR-1306-5p	14.5	25.9	2.00E-05	−0.84
ssc-miR-133a-3p	98	175	0	−0.84
ssc-miR-1343	45.7	76.8	0	−0.75
ssc-miR-145-5p	177.4	375.7	0	−1.08
ssc-miR-146b	65	133.7	0	−1.04
ssc-miR-149	11.9	19	0.00172	−0.68
ssc-miR-196b-5p	22.6	42.8	0	−0.92
ssc-miR-206	783.8	1518.8	0	−0.95
ssc-miR-214	141.1	253.2	0	−0.84
ssc-miR-296-5p	33.4	55.7	0	−0.74
ssc-miR-30b-5p	31.2	59.3	0	−0.93
ssc-miR-30c-5p	261.3	406.4	0	−0.64
ssc-miR-328	16.2	27.2	7.20E-05	−0.74
ssc-miR-423-5p	3884.5	6461.5	0	−0.73
ssc-miR-505	119.3	200.7	0	−0.75
ssc-miR-574	61.2	121.8	0	−0.99
ssc-miR-615	26.2	41.5	6.80E-06	−0.66
ssc-miR-671-3p	21	37.4	0	−0.83
ssc-miR-6782-3p	55.8	95.3	0	−0.77
ssc-miR-708-3p	42.8	72.9	0	−0.77
ssc-miR-708-5p	78.2	142.9	0	−0.87
ssc-miR-7144-5p	7.6	13.8	0.00152	−0.85
ssc-miR-874	11.5	17.3	0.00566	−0.6
ssc-miR-92b-3p	270.8	409.1	0	−0.6
ssc-miR-92b-5p	100.7	164.3	0	−0.71
ssc-miR-935	1	3.9	0.00266	−2.02
ssc-miR-99b	5940.5	9379.8	0	−0.66

Note: “|log_2_FC| = 1” means two-fold changes. Seven differentially-expressed miRNAs (DEMs) are over a two-fold change.

**Table 6:** Differential miRNA screening in D-N1ICD vs. D-Control.

miRNA	E3	E4	FDR	log_2_FC
ssc-miR-1306-3p	29.5	46.2	8E-06	−0.65
ssc-miR-132	22.9	35.8	7.00E-05	−0.64
ssc-miR-145-5p	449.1	1172.7	0	−1.38
ssc-miR-146a-5p	128.8	233.3	0	−0.86
ssc-miR-199a-3p	11555	18393	0	−0.67
ssc-miR-199b-3p	11555	18392	0	−0.67
ssc-miR-214	739.6	1459.2	0	−0.98
ssc-miR-30b-5p	38.2	84.7	0	−1.15
ssc-miR-324	1.6	4.7	0.0052	−1.55
ssc-miR-532-3p	10.3	19.4	9E-05	−0.92
ssc-miR-574	254.6	422.5	0	−0.73
ssc-miR-139-3p	6.7	3	0.0059	1.18
ssc-miR-9820-5p	6.1	2.8	0.0095	1.14

Note: “|log_2_FC| = 1” means two-fold changes. Five DEMs are over a two-fold change.

## Supplementary Materials

Supplementary materials can be found at www.mdpi.com/1422-0067/19/1/271/s1: Table S1: List of identified genes in proliferative groups (E1 and E2) and differentiative groups (E3 and E4) based on the criterion of FPKM > 2. Table S2: List of identified miRNAs in proliferative groups (S1 and S2) and differentiative groups (S3 and S4). Table S3: DEGs in proliferation phase. Table S4: Significantly-enriched GO terms of upregulated DEGs. Table S5: Significantly-enriched GO terms of downregulated DEGs. Table S6: Significantly-enriched KEGG pathways of upregulated DEGs. Table S7: Significantly-enriched KEGG pathways of downregulated DEGs. Table S8: Significantly-enriched GO terms of predicted upregulated target DEGs of downregulated DEMs. Table S9: Significantly-enriched KEGG pathways of predicted upregulated target DEGs of downregulated DEMs. Table S10: Significantly-enriched GO terms of predicted downregulated target DEGs of upregulated DEMs. Table S11: Significantly-enriched KEGG pathways of predicted downregulated target DEGs of upregulated DEMs. Table S12: Primer sequence of DEGs in P-N1ICD versus P-Control group. Table S13: Primer sequence of DEGs in D-N1ICD versus D-Control group. Table S14: Primer sequence of DEMs.

## Abbreviations

MKI67	Marker of proliferation Ki-67
WHSC1	Nuclear receptor binding SET domain protein 2
RUNX1T1	RUNX1 translocation partner 1
FGF2	Fibroblast growth factor 2
NICD	Notch intracellular domain
RBPJ	Recombination signal binding protein for immunoglobulin kappa J region
HEY	Hes related family bHLH transcription factor with YRPW motif
HEYL	Hes related family bHLH transcription factor with YRPW motif-like
PAX7	Paired box 7
P21	Cyclin dependent kinase inhibitor 1A
HES5	Hes family bHLH transcription factor 5
INHBA	Inhibin beta A subunit
SST	Somatostatin
OLR1	Oxidized low density lipoprotein receptor 1
HEY1	Hes related family bHLH transcription factor with YRPW motif 1
FLT1	Fms related tyrosine kinase 1
IL1A	Interleukin 1 alpha
TRH	Thyrotropin releasing hormone
SOCS2	Suppressor of cytokine signaling 2
VGF	VGF nerve growth factor inducible
CXCL8	C-X-C motif chemokine ligand 8
SEMA7A	Semaphorin 7A
CORO1A	Coronin 1A
BDKRB1	Bradykinin receptor B1
TMEM158	Transmembrane protein 158
DHRS3	Dehydrogenase/reductase 3
CP	Ceruloplasmin
IGF1	Insulin like growth factor 1
THPO	Thrombopoietin
EFEMP1	EGF containing fibulin extracellular matrix protein 1
WISP2	WNT1 inducible signaling pathway protein 2
CHST8	Carbohydrate sulfotransferase 8
WDR86	WD repeat domain 86
PRSS35	Protease, serine 35
C4A	Complement C4A (Rodgers blood group)
IL16	Interleukin 16
CXCL12	C-X-C motif chemokine ligand 12
SNED1	Sushi, nidogen and EGF like domains 1
AOC3	Amine oxidase, copper containing 3
AURKB	Aurora kinase B
AURKA	Aurora kinase A
PLK1	Polo like kinase 1
CDC20	Cell division cycle 20
CCNB3	Cyclin B3
ESPL1	Extra spindle pole bodies like 1, separase
CCNA2	Cyclin A2
CDC25B	Cell division cycle 25B
KIF2C	Kinesin family member 2C
CCNB1	G2/mitotic-specific cyclin-B1
THBS2	Thrombospondin 2
ACACB	Acetyl-CoA carboxylase beta
CNN1	Calponin 1
ACTG2	Actin, gamma 2, smooth muscle, enteric
EGR1	Early growth response 1
MAPK	Mitogen-activated protein kinase 1
CEBPA	CCAAT/enhancer binding protein alpha
CREB3L1	CAMP responsive element binding protein 3 like 1
ETS1	ETS proto-oncogene 1, transcription factor
ETV1	ETS variant 1
ETV4	ETS variant 4
FOSL1	FOS like 1, AP-1 transcription factor subunit
SNAI2	Snail family transcriptional repressor 2
CCNF	Cyclin F
FHOD1	Formin homology 2 domain containing 1
LRRC32	Leucine rich repeat containing 32
LIF	LIF, interleukin 6 family cytokine
ACTC1	Actin, alpha, cardiac muscle 1
FGF2	Fibroblast growth factor 2
ADM	Adrenomedullin
FGFR2	Fibroblast growth factor receptor 2 precursor
DAAM2	Dishevelled associated activator of morphogenesis 2
JAG1	Jagged 1
TIMP1	TIMP metallopeptidase inhibitor 1
VGF	VGF nerve growth factor inducible
CLU	Clusterin
TFRC	Transferrin receptor
DCN	Decorin
ALDH1A1	Aldehyde dehydrogenase 1 family member A1
ACTC1	Actin, alpha, cardiac muscle 1
SPP1	Secreted phosphoprotein 1
FGF2	Fibroblast growth factor 2
CCL5	C-C motif chemokine ligand 5
ACTG2	Actin, gamma 2, smooth muscle, enteric
HSPB7	Heat shock protein beta-7
E2F1	E2F transcription factor 1
PKMYT1	Protein kinase, membrane associated tyrosine/threonine 1
TGFB1	Transforming growth factor beta 1
TGFB3	Transforming growth factor beta 3
SKP2	S-phase kinase associated protein 2
GSI	Gamma-secretase inhibitor
CDK	Cyclin-dependent kinase
ZBTB2	Zinc finger and BTB domain containing 2
